# VisualRepbase: an interface for the study of occurrences of transposable element families

**DOI:** 10.1186/1471-2105-9-345

**Published:** 2008-08-18

**Authors:** Sébastien Tempel, Matthew Jurka, Jerzy Jurka

**Affiliations:** 1 Genetic Information Research Institute, 1925 Landings Dr., Mountain View, CA 94043, USA

## Abstract

**Background:**

Repbase is a reference database of eukaryotic repetitive DNA, which includes prototypic sequences of repeats and basic information described in annotations. Repbase already has software for entering new sequence families and for comparing the user's sequence with the database of consensus sequences.

**Results:**

We describe the software named VisualRepbase and the associated database, which allow for displaying and analyzing all occurrences of transposable element families present in an annotated genome. VisualRepbase is a Java-based interface which can download selected occurrences of transposable elements, show the distribution of given families on the chromosome, and present the localization of these occurrences with regard to gene annotations and other families of transposable elements in Repbase. In addition, it has several features for saving the graphical representation of occurrences, saving all sequences in FASTA format, and searching and saving all annotated genes that are surrounded by these occurrences.

**Conclusion:**

VisualRepbase is available as a downloadable version. It can be found at .

## Background

Text Transposable elements (TEs) are short DNA sequences (less than 25000 bp) that use different strategies to replicate and insert at different genomic locations. They can be grouped based on their mechanism of transposition. Class I mobile genetic elements, or retrotransposons, proliferate in the genome by being transcribed to RNA and then back to DNA by reverse transcriptase, while class II mobile genetic elements move directly from one position to another within the genome using a transposase to "cut and paste" them within the genome [1]. TEs can represent a large fraction of the genomic DNA in eukaryotic species. For example, more than 45% of the human genome is composed of remains of TEs [2].

Several databases have been created to collect and organize TEs. Some of them are focused on a particular family of TEs, sometimes on a single genome. For example, the Gypsy Database contains elements from the Gypsy LTR retroelement family [3], while PBmice is specialized in the piggyBac transposon in the mouse genome [4]. As far as we know, there are only four databases of TEs which are not specialized in a single family or genome: Repbase [5], MIPS Repeat Element Database (mips-REdat) [6], the Plant Repeat Database Project of TIGR [7] and TREP database [8]. Only Repbase covers TEs from all known eukaryotic species. The Plant Repeat Database Project  represents 11 plant genomes and the mips-REdat  is limited to plant genomes. The TREP database contains the transposable elements of cereal genomes. The sequences can be downloaded from all these databases and used for screening by BLAST [9], Censor [10] or RepeatMasker [11].

Repbase is a relational database that can be searched or browsed for individual TEs, which can then be downloaded in EMBL or FASTA formats [5]. New data can be contributed through the Java interface known as RepbaseSubmitter [10]. A popular version of Repbase known as RepeatMasker libraries is widely used to annotate TEs in genomic sequences [11].

Sequencing and annotation of complete eukaryotic genomes revealed the massive impact of TEs on genomic structure and evolution [2,12]. This stimulated broad interest in detailed biological studies of TEs in the genomic context. We created a specialized browser which facilitates such studies. Currently, there are browsers which allow browsing the existing annotations of TEs. Specifically, the UCSC Genome Browser [13] permits viewing of annotations of TEs and can display them at different resolutions. The annotated TEs are classified in four categories (SINEs, LINEs, LTRs and DNA transposons). However, the UCSC browser does not permit analysis of individual families and subfamilies of TEs. Another popular browser, ENSEMBL [14], has similar limitations. Moreover, these browsers do not display similarities of TEs to their consensus sequences, which is essential for dating of different layers of TE-derived repetitive elements.

## Results

### Database

We have created six tables named: Exons, Introns, Genes, NonCodingZones, Transposons and Updates. The names reflect the type of data stored in the tables. The last table stores updates of the interface and data. The first four tables were created based on genome annotations and, in addition to coordinates of the annotated sequences, they include names of the species and chromosomes. The Genes and NonCodingZones tables also specify the orientation (strand + or -), the name, and the type (gene, pseudogene, tRNA or miscRNA) of annotations. The annotations of the genomes were downloaded in the GBS/GBK format file from the NCBI website . Currently, our browser contains data for sixteen genomes, but new genome annotations will be added in the future pending demand.

The "Transposon" table was created using Censor [15], and the latest version of Repbase. It contains eight fields which include: the name of the genome, chromosome, family, superfamily, beginning and end coordinates of each TE, sequence orientation and percent of identity to the reference sequences from Repbase.

### Interface description

**Figure 1:**
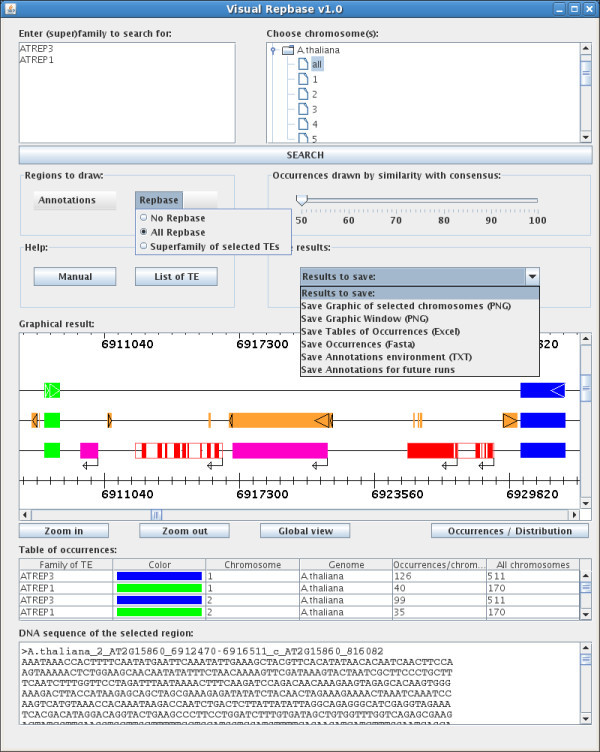
**Screenshot of VisualRepbase graphical display**. The interface contains three sections. The top section permits selection of families of TEs and eukaryotic genomes, or individual chromosomes. The middle section displays a variety of options for graphical presentation, the help menu, and output format options. The third section displays chromosomes with a graphical representation of TEs and other annotated features, tables of occurrences, and sequence information. This figure illustrates an example of two TE families from Repbase (AtREP1; AtREP3) on chromosome 2 of Arabidopsis thaliana. On the first line the selected families are displayed, including sequence orientation. On the second line other repeats from Repbase are displayed in orange, and their orientation is indicated. On the third line, other genomic annotations are displayed in addition to the selected families. The annotations include exons (in red), introns and UTRs (in framed white), and other annotated non-coding regions (in pink). Their orientations are also indicated. The table of occurrences lists the names of selected families, colors they are displayed in, chromosome name, species, number of elements on this particular chromosome, and the total number in all chromosomes displayed in the window (in this case all chromosomes of the Arabidopsis thaliana genome). The sequence is displayed in the bottom text window in FASTA format, and appears after clicking on the red rectangle which represents an exon.

VisualRepbase can be accessed using a Repbase login and password. When the user enters a valid login and password, the main interface opens. All updates will be listed in a pop-up message. The VisualRepbase interface is composed of three sections (see Figure 1).

#### Searching transposable elements

**Figure 2:**
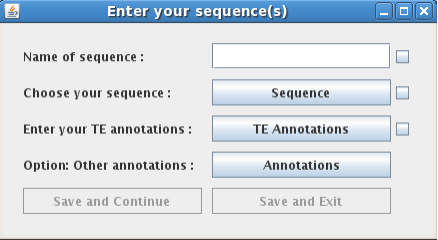
**Integration of the own sequences**. When the user clicks on the "Your Sequence(s)" icon, a new window appears. This window contains a text field where the user enters the name of his sequence and three buttons where the user enters the other information necessary for the interface. The first button selects FASTA or STADEN as the sequence format. The second button selects the transposable element annotation file. The last button selects the optional annotation file.

The first section contains the text field and the genome tree field. In the text field, the user enters the chosen name of the family or superfamily of TEs. Currently the maximum number of families one can search for at the same time is twenty. If the complete name is not known, one can type a part of the name and include an asterisk at any position. For example, typing ATREP*3, retrieves all Repbase family names beginning with "ATREP" and ending with "3" (in this case ATREP3 and ATREP13). The genome tree field permits selection of a particular genome (or genomes), and the chromosomes within that genome(s). The "all" option allows selection of all chromosomes for the given genome. The user can also enter his own sequences into the interface if he clicks on the "Your Sequence(s)" icon. A new window appears which contains a text field where the user enters the name of his sequence and three buttons where the user enters the other information necessary for the interface (Figure 2). The "SEARCH" button executes the query and the interface downloads the result of the query.

#### Visual results of the search

The second section of VisualRepbase is designed for a different type of search. It is composed of a graphical window, a table of the occurrences, and a text field displaying the sequence of the selected item. The graphical window can be split into two parts: the "occurrences graph" and the "distribution graph" of the selected biological item(s). The user can switch between these two graphs with the "Occurrences/Distribution" button (see Figure 1).

In the "occurrences graph" the orientation and position of TEs on the chromosome are displayed on different lines and each family/subfamily is displayed in a different color (see Figure 1). For each selected family/subfamily of the TEs genomic annotation can be displayed in different colors, and includes: exons in red, introns in white, all non protein coding DNA such as tRNA genes, pseudogenes and snoRNAs in pink, and the non-translated parts of the genes are in white, framed in red. The annotations of TEs are displayed in two colors: superfamilies of selected TEs in light orange and others superfamilies in orange. The orientations of the TEs and genes are indicated by pointed triangles and arrows, respectively. The interface contains three buttons for adjusting the resolution (see Figure 1). The "global view" button changes the width of the graph until the largest of the selected chromosomes is visible in the graphical window. Each click on the "Zoom in" button multiplies the chromosome width by a factor of two until the chromosome unit size reaches 100 bp. The "Zoom out" button divides the chromosome width by two, until the width of largest among the selected chromosomes is contained in the graphical window.

**Figure 3:**
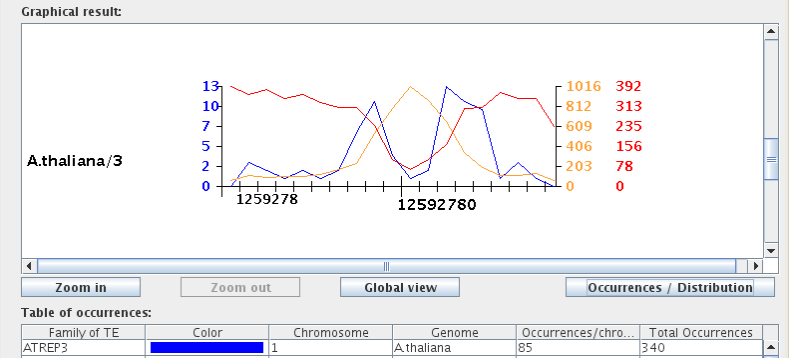
**Distribution of AtREP3 family on chromosome 3**. The graph shows the density distribution of the AtREP3 family (blue line), all RepBase TEs (orange line) and other annotations (red line), plotted against the chromosome length (x axis). The density distribution values for the selected family (ATREP3) are displayed on the left, and the corresponding numbers for other annotations are displayed on right in matching colors. The y axis is split in five intervals, and the density distribution values for the three items listed in the figure are normalized.

The "distribution graph" displays the density distribution of the selected TEs on the same chromosome(s) as the "occurrences graph", and it also permits adjustment of the view of the graphical window with the three buttons described above. Each density point corresponds to the number of occurrences within two actual units of the chromosome displayed on the screen (see Figure 3).

The table of occurrences under the graph contains six columns: "Family of TE", "Color", "Chromosome", "Genome", "Occurrences/chromosome", and "All chromosomes". Each line of this table gives the number of occurrences of a given selected TE for one chromosome and for all selected chromosomes, and gives the correspondence between the TE family and the color in the graph (see Figure 1).

The last element of the "distribution graph" is a text window which presents detailed information such as the name and position of the TE in FASTA format. Clicking on a particular biological item displays its sequence in the text window.

#### Options and saving of results

The last section of VisualRepbase presents different options for saving and drawing the interface, and the user help menu. There are two menus which display the different annotations and the TE superfamilies present for the selected chromosome. From the annotation menu, the user can choose to draw any subset of the types of annotations: protein coding genes, exons, pseudogenes, tRNA genes, miscRNAs and cis-regulatory modules. These types of annotations can be independently selected. From the Repbase menu, the user can choose to draw any Repbase family, all Repbase families, or only the superfamilies of selected TEs (see Figure 1).

The drawing option is a slide bar which displays occurrences of selected families of TEs as a function of their sequence identity to the reference sequence from Repbase.

**Figure 4:**
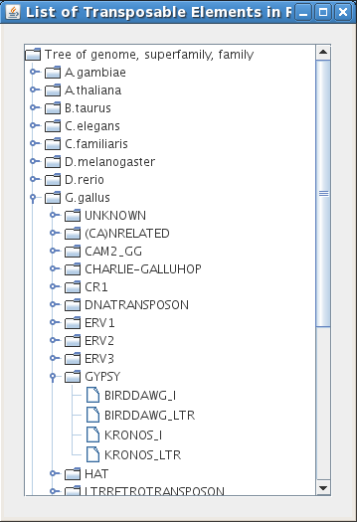
**A tree menu of transposable elements**. This menu appears after clicking on the "List of TE" button in the main menu. It corresponds to the visual tree of all families of TE present in the database. The tree permits selection by genomes, superfamilies and families.

The interface has two help buttons: "Manual" and "List of TE". The "Manual" button opens the user manual. The other button displays the list of families and subfamilies of TEs present in each genome stored in the database (see Figure 4). Clicking on the family name adds it to the list of analyzed Tes.

The "Results to Save" menu contains five different options for saving the results and one option for accelerating the future queries. The first option allows the user to save a complete picture of all chromosomes selected. The second option saves a picture of the contents visible in the graphical window at the moment that the option is selected. For both options the picture is saved in PNG format. The third option (see Figure 1) allows writing of the table of occurrences present below the graphical window to a text file. Using the fourth option one can store all sequences of TEs present in all selected chromosomes in FASTA format. The last option also permits generation of a text file containing the closest gene at the left and right of each TE and the distance between them. In some cases, the TEs are inserted in introns or they overlap with the 5' UTRs or the 3'UTRs (UnTranslated Regions) of a gene. If the TE is inserted in an intron, the software indicates that the occurrence is within the gene and it does not record the second closest gene. In the case of the 5' or 3' overlap between the TE and a gene, the software lists the overlapping gene as the closest gene on the side where the overlap occurs. The last option permits saving of all different annotations of all selected chromosomes. The interface saves them in the file called "Annot G C.vrb" where G is the name of genome and C the name of chromosome. This file is saved in the same directory in which the interface was launched. During the next run of VisualRepbase for the same chromosome, the program will use the stored information instead of the original database, which speeds up the analysis.

### Significance of the interface for biological studies

VisualRepbase can be used not only for displaying or saving the occurrences of a TE family, but it also permits studying biological problems such as the relationships between TEs and host genes. Here we give two examples illustrating its value for biological studies.

Figure 3 presents the distribution of the AtREP3 family [16] (blue), compared to all Arabidopsis TE families from Repbase (orange), and all genes on chromosome 3 from Arabidopsis thaliana. In this example, the red curve shows that the distribution of genes along chromosome 3 is uniform, except the heterochromatin centromere region, which contains less genes. In contrast, the TEs are more abundant in the heterochromatin than in the euchromatin regions (Figure 3). The distribution of the AtREP3 family is similar in the arm of the chromosome to other repeats from Repbase, but the distribution close to the centromere is very different. The distribution in the centromere is low like the distribution of the gene, but the distribution around the centromere is high. On closer inspection we found that the centromeric region is composed mostly of Gypsy-derived repeats.

**Figure 5:**
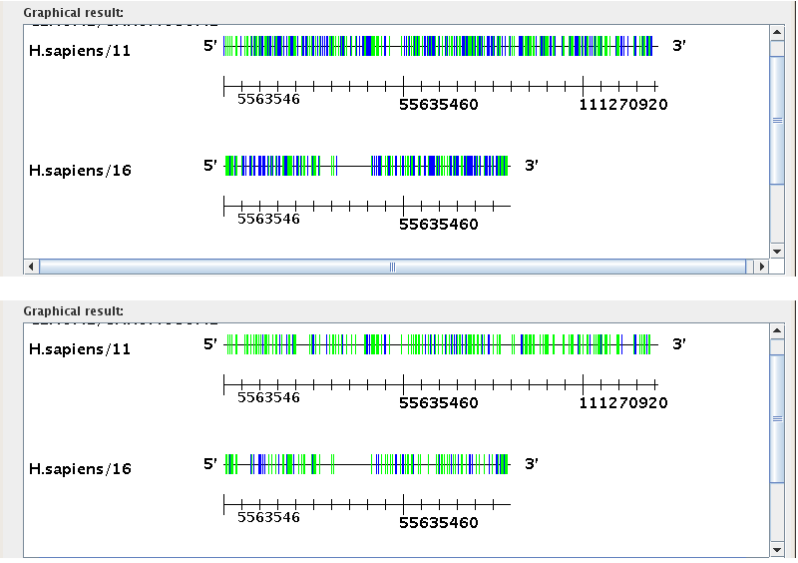
**Number of transposable elements as a function of their similarity to consensus sequences**. This figure shows the two graphs of occurrences of CHARLIE and L4 families on human chromosomes 11 and 16, for two different similarity values (50 and 75%). Each bar corresponds to one occurrence of the TE. The second graph (with 75% identity to consensus), shows that the number of L4 occurrences has decreased while the number of CHARLIE1 occurrences is similar to those in the first graph.

The visual presentation of sequence divergence also allows one to quickly estimate the age of TE families and the age difference between different families [17]. We compared occurrences of CHARLIE1 (green) and L4 (blue) (Figure 5) on human chromosomes 11 and 16 for two different sequence identity values. In Figure 5 we show the repeat distribution with 50% identity to the consensus in the first graph and higher than 75% identity in the second graph. The number of CHARLIE1 occurrences are similar in these two graphs, but the number of L4 occurrences drop dramatically from the higher range of similarities (see Figure 5). This shows that the L4 family is more divergent than the CHARLIE1 family, and it also indicates that it is older [17].

## Conclusion

VisualRepbase has been developed to facilitate studies of transposable elements in the genomic context. This is the first tool that permits a dynamic display of individual families and subfamilies in terms of their age and location in the genome. In particular, the interface permits direct visualization and comparison of older and younger layers of TE families. It facilitates inter-chromosomal and inter-genomic comparisons of TEs. VisualRepbase is also likely to stimulate studies of transposable elements associated with gene regulation.

## Availability and system requirements

Text Project name: VisualRepbase

Project home page: 

Operating system(s): Any, with Java Virtual Machine 1.4 or above

Programming language: Java

License: GPL

Any restrictions to use by non-academics: None.

## Authors' contributions

ST was responsible for programming and designing of the interface and execution of the study. MJ was responsible for the creation and update of the database and helped in the preparation of the manuscript. JJ contributed to design, supervised the study, and edited the manuscript. All authors read and approved the final manuscript.

## References

[B1] Craig N, Craigie R, M G, AM L (2002). Mobile DNA II.

[B2] Consortium IHGS (2001). Initial sequencing and analysis of the human genome. Nature.

[B3] Lloréns C, Futami R, Bezemer D, A M (2008). The Gypsy Database (GyDB) of mobile genetic elements. Nucleic Acids Res.

[B4] Sun L, Jin K, Liu Y, Yang W, Xie X, Ye L, Wang L, Zhu L, Ding S, Su Y, Zhou J, Han M, Zhuang Y, Xu T, Wu X, Gu N, Y Z (2008). PBmice: an integrated database system of piggyBac (PB) insertional mutations and their characterizations in mice. Nucleic Acids Res.

[B5] Jurka J, Kapitonov V, Pavlicek A, Klonowski P, Kohany O, Walichiewicz J (2005). Repbase Update, a database of eukaryotic repetitive elements. Cytogenet Genome Res.

[B6] Mewes H, Frishman D, Güldener U, Mannhaupt G, Mayer K, Mokrejs M, Morgenstern B, Münsterkötter M, Rudd S, B W (2002). MIPS: a database for genomes and protein sequences. Nucleic Acids Res.

[B7] Ouyang S, CR B (2004). The TIGR Plant Repeat Databases: a collective resource for the identification of repetitive sequences in plants. Nucleic Acids Res.

[B8] Wicker T, Matthews D, Keller B (2002). TREP: a database for Triticeae repetitive elements. Trends in Plant Science.

[B9] Altschul S, Gish W, Miller W, Myers E, Lipman D (1990). Basic local alignment search tool. J Mol Biol.

[B10] Kohany O, Gentles A, Hankus L, J J (2006). Annotation, submission and screening of repetitive elements in Repbase: RepbaseSubmitter and Censor. BMC Bioinformatics.

[B11] Smit A, Hubley R, Green P 1996–2006 RepeatMasker Open-3.0. http://www.repeatmasker.org.

[B12] Almeida L, Silva I, Silva WJ, Castro J, Riggs P, Carareto C, Amaral M (2007). The contribution of transposable elements to Bos taurus gene structure. Gene.

[B13] Karolchik D, Hinrichs A, Kent W (2007). The UCSC Genome Browser. Curr Protoc Bioinformatics.

[B14] Fernández-Suárez X, Schuster M (2007). Using the ensembl genome server to browse genomic sequence data. Curr Protoc Bioinformatics.

[B15] Jurka J, Klonowski P, Dagman V, P P (1996). CENSOR – a program for identification and elimination of repetitive elements from DNA sequences. Comput Chem.

[B16] Kapitonov V, Jurka J (2001). Rolling-circle transposons in eukaryotes. roc Natl Acad Sci USA.

[B17] Bergman C, Bensasson D (2007). Recent LTR retrotransposon insertion contrasts with waves of non-LTR insertion since speciation in Drosophila melanogaster. Proc Natl Acad Sci USA.

